# Socioeconomic differences in mortality amenable to health care among Finnish adults 1992-2003: 12 year follow up using individual level linked population register data

**DOI:** 10.1186/1472-6963-13-3

**Published:** 2013-01-03

**Authors:** Alison K McCallum, Kristiina Manderbacka, Martti Arffman, Alastair H Leyland, Ilmo Keskimäki

**Affiliations:** 1 Directorate of Public Health and Health Policy, NHS Lothian, Waverley Gate, 2-4 Waterloo Place, Edinburgh, EH1 3EG, Scotland; 2 National Institute for Health and Welfare, (THL), P.O. Box 30, Helsinki, FI-00271, Finland; 3 MRC/CSO Social and Public Health Sciences Unit, Lilybank Gardens, Glasgow, G12 8RZ, Scotland; 4 School of Health Sciences, University of Tampere, Kalevantie 4, Tampere, 33014, Finland

**Keywords:** Socioeconomic factors, Avoidable deaths, Equity in health care, Registers, Record linkage, Finland

## Abstract

**Background:**

Finland decentralised its universal healthcare system and introduced market reforms in the 1990s. Despite a commitment to equity, previous studies have identified persistent socio-economic inequities in healthcare, with patterns of service use that are more pro-rich than in most other European countries. To examine whether similar socio-economic patterning existed for mortality amenable to intervention in primary or specialist care, we investigated trends in amenable mortality by income group from 1992-2003.

**Methods:**

We analysed trends in all cause, total disease and mortality amenable to health care using individual level data from the National Causes of Death Register for those aged 25 to 74 years in 1992-2003. These data were linked to sociodemographic data for 1990-2002 from population registers using unique personal identifiers. We examined trends in causes of death amenable to intervention in primary or specialist healthcare by income quintiles.

**Results:**

Between 1992 and 2003, amenable mortality fell from 93 to 64 per 100,000 in men and 74 to 54 per 100,000 in women, an average annual decrease in amenable mortality of 3.6% and 3.1% respectively. Over this period, all cause mortality declined less, by 2.8% in men and 2.5% in women. By 2002-2003, amenable mortality among men in the highest income group had halved, but the socioeconomic gradient had increased as amenable mortality reduced at a significantly slower rate for men and women in the lowest income quintile. Compared to men and women in the highest income quintile, the risk ratio for mortality amenable to primary care had increased to 14.0 and 20.5 respectively, and to 8.8 and 9.36 for mortality amenable to specialist care.

**Conclusions:**

Our findings demonstrate an increasing socioeconomic gradient in mortality amenable to intervention in primary and specialist care. This is consistent with the existing evidence of inequity in healthcare use in Finland and provides supporting evidence of changes in the socioeconomic gradient in health service use and in important outcomes. The potential adverse effect of healthcare reform on timely access to effective care for people on low incomes provides a plausible explanation that deserves further attention.

## Background

Over the past fifty years, improved living conditions and comprehensive health services have reduced rates of premature death [1-3]. One aspect of health system performance is the variation in amenable mortality, premature deaths that should be avoided by timely and effective health service intervention [4]. Lists of causes of amenable mortality have been refined over time to reflect increased availability of effective interventions and the impact of health system factors on outcomes. To understand better the relationship between healthcare and amenable mortality, studies have tried to distinguish between mortality amenable to prevention, for example, immunisation, chronic disease management delivered largely in primary care, and interventions undertaken by specialist services [5-7].

Nolte and McKee found that Finland had higher amenable mortality rates than many comparable countries with only Ireland, United Kingdom and Portugal having higher rates [1]. Between 1997-8 and 2003-3 the rate fell by more than 20%. In Finland there is evidence of widespread socioeconomic differences in use of services [8-10] quality of care [9,11,12] and outcomes [11,13,14]. Equity in health care and reducing premature death from amenable causes are important objectives of health policy for all countries. Few studies, however, have examined changes in amenable mortality over time by socioeconomic status [15-17] or education [18] by type or place of intervention. Since socioeconomic inequities in access to and provision of services remain problematic, this is a significant gap in the literature.

As socio-economic differences in amenable mortality are a potential consequence of the inverse care law [19], an understanding of the context in which prevention and treatment is delivered is important. In Finland, a comprehensive healthcare system based on a network of primary healthcare centres linked to municipalities was established in 1972 [20]. The recession of the 1990s halted the expansion in primary care and preventive services [21,22] a market in the provision of primary care developed and private sector specialist ambulatory care services expanded. These services were available primarily to those in regular employment, with others reliant on municipal health centres. The recession also coincided with devolution of health service strategy to municipalities and, while there were attempts to introduce markets and commissioning with tight control over activity and budgets [23], most municipalities had limited influence over the increasing costs in specialist care. While many countries have adopted similar reforms, the Finnish experience is important for three reasons.

1) Socio-economic inequities in access to care [8,24,25] appear greater in Finland than in many other high income countries

2) Despite structural and health service changes, Finland retained substantial social welfare programmes. While income inequalities rose between 1992 and 2004 [23], they remained lower than in the United Kingdom or Sweden.

3) Welfare interventions, such as help with housing and daily living costs, reduced the potential for changes in access to social care to affect premature mortality, particularly causes amenable to primary care, despite the reduction in the income share received by those with the lowest incomes. [26].

This study analyses changes in the socioeconomic distribution of mortality amenable to primary prevention, early detection and improved treatment and medical care between 1992 and 2003 in Finland. During this period of economic recession, subsequent boom, and structural change in Finland, health service expenditure declined then slowly recovered, hospital productivity increased and patient co-payments rose [21]. Based on existing evidence of inequities in the access to and provision of services, mortality from specific causes amenable to health care and the changes in the Finnish health care system over the study period, we hypothesised that amenable mortality would be patterned by income. We also aimed to examine how the balance between mortality amenable to prevention, early intervention and treatment, and improved treatment and medical care in primary and specialist settings would change over time.

## Methods

### Classification of causes of death

Statistics Finland extracted individual data from the causes of death register for all deaths in the Finnish population aged 25-74 years between 1992 and 2003. The resident population aged 25-74 years formed the population at risk. The period studied covered the transition from ICD9 to ICD10 in 1996. Statistics Finland developed and validated procedures for forward and back translation. The completeness of death registration, the process for expert review of disputed cases and the high autopsy rate for deaths from suspicious and external causes together provide high quality mortality data [27].

### Classification of amenable mortality

We classified deaths amenable to health care based on the Nolte and McKee list [1,2]. We included premature death from chronic obstructive pulmonary disease (COPD); a category that includes chronic bronchitis and emphysema. In Finland, premature deaths classified as being primarily due to COPD are rare. Inclusion of this category reflects work undertaken by researchers in New Zealand, supported by an international expert reference group [28]. This reflects recognition that, while COPD is largely preventable by being a non-smoker, effective interventions available in primary care include active chronic disease management: prevention of exacerbations through smoking cessation and immunization against influenza and pneumococcal disease, rapid treatment and pulmonary rehabilitation. We analysed deaths from ischaemic heart disease separately. To take account of the small number of deaths from individual conditions, we grouped them by whether they were mainly amenable to individual preventive services, early detection and intervention, and improved treatment and medical care [5]. We further subdivided the improved treatment and medical care group into whether treatment was undertaken mostly in primary or specialist care [2,29,30] (see Additional file 1: Appendix 1).

We included conditions amenable to primary prevention by the health care system such as infections that are preventable by hygiene measures or by immunisation. Although these interventions have a population-level impact, individual patients receive these services from primary care. Deaths amenable to early detection and intervention include tuberculosis; patients with symptoms of these conditions require rapid access to diagnosis and treatment. Similarly, while not all premature deaths from hypertension, cerebrovascular disease or cancers with a detectable pre-malignant stage are preventable, the amenable mortality rate can be reduced by prompt identification and effective, protocol based intervention. Mortality amenable to improved treatment and medical care comprises those conditions where the likelihood of death without treatment is high and includes diabetes and thyroid disease (where most treatment is in primary care). These are chronic diseases for which a combination of medication, active follow-up and secondary prevention reduces the risk of adverse events. By comparison, conditions amenable to specialist care include cancers without an obvious preventable cause such as cancer of the testis, where treatment in a specialist centre produces high cure rates.

We ran the analyses for all cause, total disease and amenable mortality. We did not categorise separately deaths amenable to health policy, such as those related to substance misuse. We were unable to include deaths due to “accident or misadventure due to healthcare” as these are not classified separately in Finnish routine datasets on causes of death. Such deaths usually represent a small fraction of all amenable mortality [2].

### Income data

We studied amenable mortality by family net income in adults (25-74 years). Income data from annual tax statistics were linked to individuals by personal identification codes. To avoid indirect identification of rare events, Statistics Finland tabulated annual data by socio-demographic variables. Age was classified into five year age bands and income was adjusted for family size using the OECD equivalence scale and grouped according to quintile limits derived from the population at risk [30]. Those with no recorded income were assigned to the lowest income group. Income was linked to individuals based on income during the previous calendar year for the entire population at risk.

### Statistical methods

We directly age-standardized mortality rates in 1992-93 and 2002-03 for total and subgroups of amenable mortality using the European standard population. Annual age-standardized mortality rates were calculated by income quintile. We examined trends in amenable mortality by income quintiles using a repeated cross-sectional design, with the count of deaths in cells defined by income quintile, age group and year modelled using Poisson regression and including an interaction between income and year. All models were stratified by sex.

#### *Trends*

While the main focus was of our work was on relative inequalities, we examined trends over time in the absolute levels of socioeconomic patterning of amenable mortality by reviewing the numbers of deaths, evaluating changes in the age-standardised amenable mortality rate and calculating the reduction in the average amenable mortality rate by income quintile. Average annual changes were obtained from estimates of linear time trends in age-adjusted repeated measures Poisson regression models.

To assess differences in amenable mortality by income quintile we examined data in three four-year periods (1992-1995, 1996-1999 and 2000-2003). Again, we modelled mortality rates using Poisson regression models but with these periods in place of the individual year. Rate ratios for contrasts were used to describe the relative magnitude of amenable mortality in lower income quintiles compared to the highest one in each of the three periods [31]. Further, we determined the annual contribution of mortality amenable to the place of intervention: primary and specialist care, and to the type of intervention: individual preventive services, early detection and intervention, and improved treatment and medical care to differences in total amenable mortality between the highest and lowest income quintiles. To minimise potential misclassification of income or amenability associated with serious underlying disease, we also analysed income differences excluding patients resident in long-term institutions.

Statistical analyses were performed using SAS System for Windows, release 9.1.3. [32].

## Results

During the study period, the total number of deaths in people aged 25-74 years declined from a two year average of 21609 in 1992-93 to a two year average of 18719 in 2002-03. Total disease mortality (excluding external causes) reduced from 18430 to 15994. The number of deaths per year classified as amenable among men and women aged 25 to 74 years decreased from a two year average of 4072 in 1992-1993 to 3265 in 2002-2003. Table 1 illustrates the reduction in all cause, total disease and amenable mortality.

**Table 1:** Age-standardized amenable mortality rate for 100,000 population aged 25-74, proportion of total amenable mortality (years 1992-93 and 2002-03) and average annual changes (%)

	**1992-93**			**2002-03**			**Average annual change**	
	**avg number of deaths per year**	**Mortality rate**	**% of total amenable mortality**	**avg number of deaths per year**	**Mortality rate**	**Amenable mortality**	**%**	**95%CI**
**Men**								
All cause mortality	14038	990		12512	739		-2.8	( -3.0 - -2.7)
Total disease mortality	11557	828		10395	611		-3.0	( -3.1 - -2.8)
Total amenable mortality	2032	144	100	1678	99	100	-3.6	( -4.0 - -3.2)
Primary prevention	0	0	0.1	0	0		*	
Early detection and intervention	1358	97	67.7	1137	67	67.6	-3.7	( -4.2 - -3.3)
- tuberculosis	40	3	2.0	18	1	1.0	-10.0**	(-13.1 - -6.9)
- malignant neoplasm	273	20	13.7	289	17	17.2	-1.3	( -2.2 - -0.3)
- hypertension and cerebrovascular disease	1046	75	52.0	831	49	49.3	-4.3	( -4.8 - -3.8)
Improved treatment and medical care	674	46	32.3	541	32	32.4	-3.3	( -3.9 - -2.6)
- Predominantly primary care	105	6	4.4	84	5	5.1	-1.3	( -3.1 - 0.4)
- Predominantly specialist care	569	40	27.9	457	27	27.3	-3.6	( -4.3 - -2.9)
Ischaemic heart disease	4188	303		2926	172		-5.6	( -5.9 - -5.3)
**Women**								
All cause mortality	7571	420		6207	324		-2.6	( -2.8 - -2.4)
Total disease mortality	6873	377		5599	289		-2.6	( -2.8 - -2.4)
Total amenable mortality	2040	114	100	1587	83	100	-3.1	( -3.5 - -2.7)
Primary prevention	0	0		0	0		*	
Early detection and intervention	1653	93	81.4	1320	69	83.0	-3.0	( -3.5 - -2.6)
- tuberculosis	22	1	1.0	9	0	0.5	-6.7**	(-10.7 - -2.7)
- malignant neoplasm	782	47	40.8	756	40	48.7	-1.0	( -1.6 - -0.4)
- Hypertension and cerebrovascular disease	849	45	39.6	556	28	33.8	-5.4	( -6.0 - -4.7)
Improved treatment and medical care	387	21	18.6	267	14	17.0	-3.2	( -4.1 - -2.3)
- Predominantly primary care	49	3	2.6	43	3	3.1	-1.1	( -3.5 - 1.3)
- Predominantly specialist care	338	18	15.9	224	12	13.9	-3.5	( -4.5 - -2.6)
Ischaemic heart disease	1573	80		844	41		-6.8	( -7.3 - -6.4)

* Due to small numbers RRs were not estimated.

** Due to few deaths in younger age bands 25-44 years old were combined into one age-group for RR estimates.

Amenable mortality decreased more rapidly than all cause and total disease mortality. Among men in 2002-03, amenable mortality accounted for 13% of total mortality and 16% of disease mortality, among women the figures were 26% and 28%. The average annual reduction in amenable mortality (3.6%) was greater than that for all cause (2.8%) and total disease mortality (3.0%) for men. Among women the reduction was more modest. The annual average decline in amenable mortality across the study period was 3.1%, compared with 2.6% for all cause and for total disease mortality. The annual average decline in amenable mortality showed a socioeconomic gradient; in the highest income group this was -5.9% (95% CI -6.7 to -5.8) for men and -4.1% (95% CI -5.1 to -3.1) for women, whereas in the lowest income group the annual average decline was -0.9% (95% CI -2.0 to 0.2) for men and -1.0 (95% CI -2.3 to -0.3) for women.

The largest subgroups of amenable mortality comprised conditions suitable for early detection and intervention (66% of total amenable mortality among men in 1992-93, and 80% among women). Mortality amenable to improved treatment and medical care comprised 33% of amenable deaths among men and 20% among women. By 2002-2003, deaths amenable to improved treatment and medical intervention in primary care comprised 5% of amenable mortality among men and 3% among women while deaths responsive to intervention in secondary care accounted for 29% of amenable mortality among men and 17% among women.

In 1992-93, the start of our study period, ischaemic heart disease (IHD) comprised 30% of total mortality and 36% of disease mortality among men aged 25-74 years. Among women, IHD mortality accounted for 19% of total mortality and 22% of disease mortality. Between 1992-2003 the annual number of ischaemic heart disease deaths declined from 5761 to 3771, from 192 to 109 per 100,000 among men, and from 51 to 26 per 100,000 in women. The average annual reduction in mortality attributed to ischaemic heart disease was 5.6% in men (95% CI 5.9 to -5.3%) and 6.8% among women (7.3 to -6.4%).

All income groups and both genders experienced an average annual reduction in premature mortality attributable to ischaemic heart disease that was greater than that for amenable mortality excluding IHD. The average annual reduction was -7.9 (95% 8.6 to 7.1) among men and -7.2 (95% CI -9.7 to -4.7) among women in the highest income group compared with -2.3 (95% CI -3.2 to -1.5) among men and -3.8 (95% CI -4.8 to -2.8) among women in the lowest income group.

Tables 2 (men) and 3 (women) present the gradient in risk ratios for amenable mortality by income in the three periods. The annual rate of change appeared fairly linear and while the pattern differed by income group, none of the rules for using this approach were violated. Among men, the gradient in amenable mortality across income groups was similar to that found in all cause and total disease mortality. The gradients increased from the early 1990s to early 2000s. The rate ratios by income in mortality amenable to early detection and intervention were more modest but followed a similar trend, increasing from 2.00 in 1992-3 to 2.66 in 2002-3. Relative income differences were considerably larger for mortality amenable to improved treatment and medical care than those found for all cause (2.53 in 1992-3 to 3.26 in 2002-3) or disease mortality in the lowest income group (1.20 (not significant) in 1992-3 to 2.91 in 2002-2003). The gradient in mortality amenable to improved treatment and medical care also increased over the study period. In 1992-3, the risk ratio for mortality amenable to improved treatment and medical care in primary care was 5.87 and 5.31 for treatment in specialist settings. By 2002-03, the relative mortality risk of men in the lowest income group was 14 times that of the highest income group for mortality amenable to primary care and just less than nine times for mortality amenable to specialist care.

**Table 2:** Socioeconomic gradients in risk ratios for amenable mortality by income and cause of death, men aged 25-74

				**Period I: 1992 – 95**		**Period II: 1996 - 99**		**Period III: 2000 - 03**		**Period interactions**	
	**Income**	**RR**	**95% CI**	**RR**	**95% CI**	**RR**	**95% CI**		**p value**	
All cause mortality									
	5	1.00		1.00		1.00		PI vs PII	<.001
	4	1.35	(1.18 - 1.54 )	1.39	(1.20 - 1.61 )	1.41	(1.23 - 1.61 )	PII vs PIII	<.001
	3	1.67	(1.49 - 1.88 )	1.77	(1.55 - 2.02 )	1.80	(1.58 - 2.04 )	PI vs PIII	<.001
	2	2.15	(1.89 - 2.44 )	2.41	(2.10 - 2.77 )	2.60	(2.26 - 3.00 )		
	1	2.89	(2.54 - 3.28 )	3.49	(3.03 - 4.03 )	4.17	(3.57 - 4.88 )		
Total disease mortality									
	5	1.00		1.00		1.00		PI vs PII	<.001
	4	1.36	(1.19 - 1.57 )	1.41	(1.22 - 1.64 )	1.42	(1.24 - 1.63 )	PII vs PIII	<.001
	3	1.67	(1.47 - 1.89 )	1.77	(1.54 - 2.03 )	1.81	(1.59 - 2.06 )	PI vs PIII	<.001
	2	2.07	(1.81 - 2.38 )	2.32	(2.01 - 2.769)	2.53	(2.17 - 2.94 )		
	1	2.59	(2.28 - 2.95 )	3.18	(2.73 - 3.71 )	3.82	(3.23 - 4.52 )		
Total amenable mortality									
	5	1.00		1.00		1.00		PI vs PII	<.001
	4	1.20	(0.99 - 1.45 )	1.46	(1.19 - 1.78 )	1.35	(1.10 - 1.66 )	PII vs PIII	0.09
	3	1.46	(1.23 - 1.73 )	1.68	(1.42 - 1.99 )	1.65	(1.39 - 1.97 )	PI vs PIII	<.001
	2	1.90	(1.59 - 2.267)	2.31	(1.91 - 2.80 )	2.43	(1.98 - 2.97 )		
	1	2.83	(2.40 - 3.33 )	3.91	(3.27 - 4.68 )	4.13	(3.37 - 5.06 )		
Early detection and intervention									
	5	1.00		1.00		1.00		PI vs PII	<.001
	4	1.11	(0.94 - 1.29 )	1.37	(1.18 - 1.60 )	1.28	(1.08 - 1.53 )	PII vs PIII	0.49
	3	1.31	(1.14 - 1.50 )	1.53	(1.35 - 1.74 )	1.47	(1.27 - 1.70 )	PI vs PIII	<.001
	2	1.61	(1.40 - 1.85 )	1.87	(1.61 - 2.19 )	1.98	(1.66 - 2.35 )		
	1	2.00	(1.75 - 2.27 )	2.67	(2.26 - 3.16 )	2.66	(2.25 - 3.15 )		
- malignant neoplasm									
	5	1.00	*	1.00		1.00		PI vs PII	0.09
	4	0.85	(0.66 - 1.08 )	1.10	(0.94 - 1.28 )	1.05	(0.85 - 1.31 )	PII vs PIII	0.05
	3	0.78	(0.63 - 0.97 )	1.12	(0.97 - 1.29 )	1.02	(0.85 - 1.21 )	PI vs PIII	0.46
	2	0.98	(0.79 - 1.21 )	1.18	(1.03 - 1.35 )	1.21	(1.03 - 1.41 )		
	1	0.90	(0.75 - 1.08 )	1.25	(1.01 - 1.55 )	1.31	(1.04 - 1.64 )		
- hypertension and cerebrovascular disease									
	5	1.00		1.00		1.00		PI vs PII	<.001
	4	1.21	(1.03 - 1.43 )	1.51	(1.27 - 1.79 )	1.41	(1.13 - 1.76 )	PII vs PIII	0.25
	3	1.52	(1.32 - 1.75 )	1.71	(1.49 - 1.97 )	1.72	(1.43 - 2.05 )	PI vs PIII	<.001
	2	1.85	(1.60 - 2.15 )	2.19	(1.85 - 2.59 )	2.39	(1.94 - 2.96 )		
	1	2.37	(2.06 - 2.72 )	3.23	(2.72 - 3.84 )	3.35	(2.75 - 4.07 )		
-Improved treatment and medical care									
	5	1.00		1.00		1.00		PI vs PII	<.001
	4	1.46	(1.07 - 1.97 )	1.69	(1.20 - 2.39 )	1.57	(1.18 - 2.08 )	PII vs PIII	0.03
	3	1.87	(1.41 - 2.48 )	2.09	(1.50 - 2.21 )	2.25	(1.74 - 2.92 )	PI vs PIII	<.001
	2	2.77	(2.06 - 3.71 )	3.74	(2.75 - 5.09 )	4.01	(3.06 - 5.26 )		
	1	5.47	(4.16 - 7.19 )	7.93	(5.93 - 10.6 )	9.43	(7.32 - 12.1 )		
- predominantly primary care									
	5	1.00		1.00		1.00		PI vs PII	0.07
	4	1.95	(1.29 - 2.93 )	1.54	(1.02 - 2.32 )	2.14	(1.18 - 3.88 )	PII vs PIII	0.06
	3	2.61	(1.84 - 3.72 )	2.18	(1.39 - 3.43 )	4.01	(2.32 - 6.93 )	PI vs PIII	<.001
	2	3.90	(2.79 - 5.44 )	5.07	(3.20 - 8.02 )	6.66	(3.84 - 11.5 )		
	1	5.87	(4.00 - 8.62 )	7.84	(5.09 - 12.0 )	14.00	(8.25 - 23.8 )		
- predominantly specialist care									
	5	1.00		1.00		1.00		PI vs PII	<.001
	4	1.35	(0.97 - 1.87 )	1.71	(1.19 - 2.45 )	1.47	(1.08 - 2.01 )	PII vs PIII	0.06
	3	1.73	(1.27 - 2.34 )	2.05	(1.42 - 2.95 )	2.04	(1.56 - 2.67 )	PI vs PIII	<.001
	2	2.57	(1.88 - 3.51 )	3.54	(2.56 - 4.90 )	3.69	(2.80 - 4.87 )		
	1	5.31	(3.97 - 7.11 )	7.95	(5.88 - 10.8 )	8.77	(6.76 - 11.4 )		
Ischaemic heart disease (IHD)									
	5	1.00		1.00		1.00		PI vs PII	0.115*
	4	1.59	( 1.19 -2.12)	1.42	( 1.12 - 1.81)	1.52	( 1.10 - 2.09)	PII vs PIII	0.002
	3	2.04	( 1.56 - 2.67)	1.90	( 1.51 - 2.39	1.93	( 1.46 - 2.55)	PI vs PIII	0.001
	2	2.54	( 1.93 - 3.32)	2.58	( 2.10 - 3.17)	2.95	( 2.17 - 4.01)		
	1	3.51	( 2.66 - 4.62)	3.90	( 3.13 - 4.86)	4.74	( 3.44 - 6.52)		

* linear income trend not significant at 95% significance level.

Socioeconomic differences in amenable mortality by income and cause of death, men aged 25-74.

**Table 3:** Socioeconomic gradients in risk ratios for amenable mortality by income and cause of death, women aged 25-74

		**Period I: 1992 - 95**		**Period II: 1996 - 99**		**Period III: 2000 - 03**		**Period interactions**	
	**Income**	**RR**	**95% CI**	**RR**	**95% CI**	**RR**	**95% CI**		**P value**
All cause mortality									
	5	1.00		1.00		1.00		PI vs PII	<.001
	4	1.27	(1.09 - 1.47 )	1.28	(1.10 - 1.48 )	1.29	(1.12 - 1.49 )	PII vs PIII	0.12
	3	1.52	(1.34 - 1.73 )	1.55	(1.36 - 1.76 )	1.54	(1.34 - 1.77 )	PI vs PIII	<.001
	2	1.82	(1.60 - 2.06 )	1.98	(1.74 - 2.25 )	2.00	(1.70 - 2.35 )		
	1	2.53	(2.24 - 2.86 )	2.98	(2.63 - 3.38 )	3.26	(2.68 - 3.96 )		
Total disease mortality									
	5	1.00		1.00		1.00		PI vs PII	<.001
	4	1.29	(1.11 - 1.50 )	1.29	(1.11 - 1.49 )	1.30	(1.14 - 1.48 )	PII vs PIII	0.24
	3	1.53	(1.34 - 1.74 )	1.55	(1.37 - 1.75 )	1.53	(1.34 - 1.75 )	PI vs PIII	<.001
	2	1.80	(1.58 - 2.05 )	1.93	(1.71 - 2.18 )	1.95	(1.67 - 2.27 )		
	1	2.47	(2.18 - 2.81 )	2.91	(2.58 - 3.28 )	3.12	(2.58 - 3.76 )		
Total amenable mortality									
	5	1.00		1.00		1.00		PI vs PII	<.001
	4	1.19	(1.04 - 1.38 )	1.19	(1.04 - 1.38 )	1.24	(1.12 - 1.38 )	PII vs PIII	0.05
	3	1.37	(1.22 - 1.55 )	1.43	(1.25 - 1.64 )	1.47	(1.31 - 1.65 )	PI vs PIII	<.001
	2	1.52	(1.34 - 1.72 )	1.62	(1.42 - 1.85 )	1.77	(1.56 - 2.01 )		
	1	2.22	(1.97 - 2.51 )	2.57	(2.26 - 2.92 )	2.93	(2.53 - 3.40 )		
Early detection and intervention									
	5	1.00		1.00		1.00		PI vs PII	0.04
	4	1.17	(1.04 - 1.32 )	1.19	(1.06- 1.35 )	1.20	(1.08 - 1.34 )	PII vs PIII	0.17
	3	1.33	(1.20 - 1.46 )	1.39	(1.23 - 1.57 )	1.38	(1.23 - 1.55 )	PI vs PIII	<.001
	2	1.40	(1.27- 1.54)	1.49	(1.32 - 1.67 )	1.58	(1.41 - 1.76 )		
	1	1.80	(1.63 - 1.99 )	1.99	(1.73- 2.29 )	2.19	(1.93 - 2.48 )		
- malignant neoplasm									
	5	1.00	*	1.00		1.00		PI vs PII	0.01
	4	1.11	(0.96 - 1.29 )	1.19	(1.01 - 1.40 )	1.13	(0.96 - 1.32 )	PII vs PIII	<.001
	3	1.23	(1.08 - 1.39 )	1.40	(1.98 - 1.65 )	1.31	(1.09 - 1.56 )	PI vs PIII	<.001
	2	1.10	(0.96 - 1.26 )	1.28	(1.08 - 1.52 )	1.37	(1.14 - 1.63 )		
	1	1.06	(0.91 - 1.24 )	1.29	(1.10 - 1.52 )	1.57	(1.31 - 1.87 )		
- hypertension and cerebrovascular disease									
	5	1.00		1.00		1.00		PI vs PII	0.11
	4	1.29	(1.07 - 1.57 )	1.23	(1.01 - 1.51 )	1.49	(1.32 - 1.66 )	PII vs PIII	0.21
	3	1.55	(1.31 - 1.82 )	1.46	(1.26 - 1.71 )	1.74	(1.57 - 1.94 )	PI vs PIII	0.01
	2	1.87	(1.62 - 2.15 )	1.92	(1.69 - 2.18 )	2.26	(2.01 - 2.53 )		
	1	2.83	(2.46 - 3.26 )	3.16	(2.72 - 3.66 )	3.75	(3.15 - 4.45 )		
Improved treatment and medical care									
	5	1.00		1.00		1.00		PI vs PII	<.001
	4	1.35	(0.91 - 2.01 )	1.14	(0.73 - 1.77 )	1.71	(1.11 - 2.64 )	PII vs PIII	<.001
	3	1.71	(1.17 - 2.49 )	1.73	(1.15 - 2.61 )	2.46	(1.82 - 3.31 )	PI vs PIII	<.001
	2	2.41	(1.66 - 3.51 )	2.60	(1.76 - 3.83 )	4.02	(2.90 - 5.56 )		
	1	5.44	(3.79 - 7.81 )	6.70	(4.55 - 9.88 )	11.41	(8.15 - 16.0 )		
- predominantly primary care									
	5	1.00		1.00		1.00		PI vs PII	0.41
	4	1.20	(0.69 - 2.07 )	1.52	(0.91 - 2.55 )	2.14	(1.08 - 4.23 )	PII vs PIII	<.001
	3	1.56	(0.89 - 2.73 )	1.99	(1.17 - 3.38 )	3.43	(1.79 - 6.57 )	PI vs PIII	<.001
	2	2.85	(1.60 - 5.07 )	3.55	(2.18 - 5.80 )	8.42	(4.33 - 16.4 )		
	1	4.55	(2.79 - 7.42 )	6.01	(3.88 - 9.33 )	20.54	(11.4 - 36.8 )		
- predominantly specialist care									
	5	1.00		1.00		1.00		PI vs PII	0.01
	4	1.40	(0.90 - 2.18 )	1.06	(0.65 - 1.73 )	1.66	(1.05 - 2.62 )	PII vs PIII	0.06
	3	1.74	(1.14 - 2.66 )	1.70	(1.07 - 2.71 )	2.32	(1.70 - 3.17 )	PI vs PIII	<.001
	2	2.37	(1.55 - 3.62 )	2.51	(1.64 - 3.81 )	3.58	(2.56 - 5.02 )		
	1	5.62	(3.72 - 8.49 )	6.90	(4.50 – 10.6 )	10.36	(7.14 - 15.0 )		
Ischaemic heart disease									
	5	1.00		1.00		1.00		PI vs PII	<.001
	4	1.40	(1.24-1.58)	1.49	(1.30-1.70)	1.57	(1.39-1.78)	PII vs PIII	<.001
	3	1.71	(1.54-1.90)	1.91	(1.67-2.18)	2.01	(1.77-2.29)	PI vs PIII	<.001
	2	2.15	(1.92-2.40)	2.51	(2.18-2.90)	2.81	(2.43-3.25)		
	1	2.51	(2.27-2.80)	2.97	(2.61-3.39)	4.01	(3.51-4.59)		

* linear income trend not significant at 95% significance level.

Socioeconomic differences in amenable mortality by income and cause of death. women aged 25-74.

Among women, the income gradient was generally less pronounced than for men although exceptions were seen for mortality amenable to improved treatment and medical care. Here the rate ratio in the lowest income group was 20.54 times that of the highest income group for primary care and 10.36 for specialist care by 2000-2003.

There was no significant gradient for cancer at the start of the study period. The gradient remained modest relative to other conditions but it increased to 1.31 for men and 1.57 for women by 2003. While the mix of cancers was different for men and women, cancer is an important source of amenable mortality for women comprising 40% in 1992, rising to 48% of total amenable mortality by 2003.

The gradient in premature mortality attributed to ischaemic heart disease was similar to that found when all categories of amenable mortality were combined. As was found for amenable mortality, the income gradient for ischaemic heart disease increased significantly, albeit more modestly, over time (p < 0.01).

**Figure 1:**
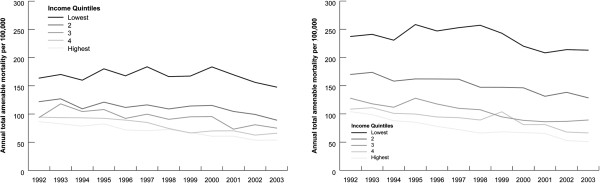
**Amenable mortality rates for 25-74 years old by income quintiles in 1992-2003.** See Additional file 1: Table S1

Figure 1 shows that amenable mortality declined in men and women in all income groups. By 2003 the gender gap had disappeared for those in the highest and second highest income group. Overall amenable mortality in men in the highest income group (50.98 per 100,000) was lower than that for women (53.85 per 100,000). The excess amenable mortality in men persisted for those in the lowest income group; in 2003 the rate in men was 1.44 times that in women. The decline in amenable mortality in men and women in the lowest income group between 1992-2003 was modest, around 11% (from 237.23 per 100,000 in 1992 to 212.96 in 2003), while the rate halved for men in the highest income group (from 103.99 in 1992 50 50.98 in 2003). Reanalysing the data excluding individuals resident in long term institutions decreased income gradients a little, but did not alter the findings.

## Discussion

Analysis of comprehensive, individual level data demonstrated that amenable mortality in Finland varied systematically by income group between 1992 and 2003: the lower the income, the higher the risk of amenable mortality. During this period, the absolute and relative gap between the highest and lowest income groups widened with an excess of 28.74 amenable deaths per 100,000 among men in 2003 compared with 1992. Income related differences were particularly high for mortality amenable to improved treatment and medical care in primary healthcare. Towards the end of the study period, the risk ratio for amenable mortality for the lowest income quintile was 14 times that of the highest quintile for men and 21 times that for women. These large risk ratios reflect very few deaths from causes such as asthma, diabetes or epilepsy among the richest quintile.

The socio-economic gradient in amenable mortality increased, with limited reductions in the age-standardized death rates among the lowest income group. Widening inequalities in all cause and total disease mortality were more modest. While a detailed analysis of gender differences in amenable mortality is beyond the scope of this paper, amenable mortality rates remained higher in men than women except for the highest income quintile. In addition, the gender gap for amenable mortality was narrower than that for all-cause or total disease mortality and the reduction over time greater [33].

Since the initial studies of amenable mortality, the age limit for amenable death has increased from 65 to 75 years in Western Europe, reflecting increased life expectancy [1]. The fall in premature deaths reflects lower exposure to several risk factors, notably smoking, and more effective medical care [34,35]. Despite the overall decline in amenable mortality rates [36,37] the differential reductions by income groups and the persistent socio-economic gradient illustrates the role of the health system in perpetuating health inequalities [15,38]. Furthermore, the gap between the lowest and second lowest socio-economic group, or between those inside and outside of the workforce [16,39] suggests that health services are not reaching the most disadvantaged. Although the rate is smaller in other countries, the pattern of excess amenable mortality due to chronic conditions such as diabetes is similar to that found among in lower income and minority ethnic groups elsewhere [7,15-18]. More detailed analysis of the relationship between amenable mortality rates and inequities in access, quality and comprehensiveness of care is warranted.

### Strengths and limitations

We applied current definitions of amenable mortality [2,7] but our findings are based on comprehensive and reliable individually based linked register data. The level of confirmation of diagnosis at death by autopsy in Finland is high by international comparison [40].

Our data also enabled us to consider the impact of health service changes in the risk of amenable mortality by socioeconomic group. In many studies, it is difficult to determine the impact of changes in non-IHD mortality. We analysed IHD deaths separately for three reasons. Firstly, because of the continuing debate about the proportion of mortality from IHD that is amenable to health service intervention. Secondly, the large number of IHD deaths masks the pattern of amenable deaths from other causes, and thirdly, because we analysed individual level, not aggregate data, we could not exclude a proportion of deaths from one condition. We were, however, able to compare trends in IHD deaths with those for amenable mortality as a whole and with other causes of amenable mortality.

Data were incomplete in three areas. As with other register based studies, we had no behavioural data although alcohol and tobacco use clearly contribute to the socio-economic gradient in premature deaths [41,42]. While the major contribution to reducing tobacco and alcohol related deaths results from policy interventions, smoking cessation [43] and alcohol brief interventions [44] are evidence based primary care treatments that could be used widely among lower income groups in Finland, but are not [45,46]. We also omitted healthcare-associated deaths, as Statistics Finland does not identify these separately in the Causes of Death register.

While we have access to high quality linked data, and we checked the classification of deaths as amenable was appropriate, researchers and policy makers continue to debate the inclusion and exclusion of specific causes, the relationship between evidence for intervention and impact on amenable mortality rates and the boundary between health care and interventions excluded because they are classified as multi-agency or health promotion. All of these concerns are legitimate and are reflected in the final report of the AMIEHS project : Avoidable mortality in the European Union:

Towards better indicators for the effectiveness of health systems. (http://amiehs.lshtm.ac.uk/publications/reports/AMIEHS%20final%20report%20VOL%20I.pdf). We agree with the authors’ caution to avoid direct attribution of a causal relationship between deaths from amenable causes and health system effectiveness but consider that our findings are worrying and worthy of further investigation.

With this dataset, we were also unable to adjust for socio-economic differences in incidence, in the treatability of disease associated with co-morbidity, or delays in seeking medical care. In a universal healthcare system, differential changes in the incidence of treatable disease should not affect the socio-economic gradient in amenable mortality by virtue of being treatable. The extent of the socioeconomic gradient in multiple morbidity and its earlier onset in primary care patients living in areas of multiple deprivation has only recently been recognized [47]. It seems plausible that the gradient in amenable mortality reflects the earlier onset and treatability of multiple morbidity in a less responsive health care environment rather than a rise in the incidence of a single specific cause. There is also evidence that lower socio-economic groups have more limited access to treatment known to reduce the risk of early death [48-50] even after adjustment for individual clinical characteristics. However, there is some evidence that attention to populations with higher incidence of disease in designing programmes of care can reduce some aspects of amenable mortality, for example survival after myocardial infraction in South Asian populations in Scotland [51]. Attention to some of the previously identified socioeconomic inequities in access to secondary prevention and treatment of ischaemic heart disease, including revascularization, may explain the more modest increase in gradient in the lowest income group. Similarly, data from the UK indicate that investing in common, chronic conditions associated with amenable mortality can improve outcome [52] although further work is necessary to identify the optimal model of care to address the socioeconomic and age gradient in multiple morbidity.

Since this was a cross-sectional study relating mortality to current income, we were unable to infer the direction of causation. Changes over time in the selection of people with progressive illnesses and multiple morbidity into lower income quintiles could partly explain the gradients found.

### Structural change in Finnish health services

Access to diagnosis and ongoing treatment regardless of socioeconomic status or geography characterises universal health systems. However gradients in amenable mortality, particularly for conditions such as hypertension and diabetes persist [7,15,16,38,50]. At the start of the study period, the 2-3 fold socioeconomic gradient in amenable mortality in Finland was similar to that reported by other comprehensive healthcare systems, despite differences in funding, generosity of the social welfare system, and study methodology. By the early 2000s in Finland, educational inequalities in amenable mortality were greater than in other Nordic countries [18] and income related differences were substantially larger.

Inequalities in amenable mortality in Finland widened during a period of structural and economic change. During the study period, municipal primary care received relatively less investment than specialist services. Since the late 1990s most people in stable employment receive primary healthcare from providers with which their employer contracts and GPs no longer provide comprehensive primary care. The expansion in private specialist ambulatory care services also provides more affluent groups with additional opportunities to access care and have the costs partially reimbursed by the social insurance system. The design of these newer services may have changed the help-seeking behaviour of men in higher income groups. Our findings may also reflect the differential impact of co-morbidity and complexity of need on the ability of people with lower income to access acute and chronic care in an increasingly complex Finnish healthcare system.

While most chronic conditions occur more frequently among lower income groups, rising co-payments and limited exemptions associated with the health service reforms increase the financial burden of healthcare [53]. In addition, the Finnish health system has been slow to adopt active methods of improving access and treatment [54] for patients with chronic conditions in primary care. Disease registers, call-recall systems, outreach services, continuity of personal care, patient involvement in assessing need and designing services are not widespread in Finland.

Recent analysis of empirical and experimental research has identified some interventions that may reduce socio-economic inequalities in access to healthcare [55,56]. These include abolition of user fees, strengthening primary care so that it is universally available, expert-based, and actively engages people from disadvantaged groups, particularly those with chronic conditions.

## Conclusions

The pattern of amenable deaths in Finland for conditions amenable to treatment and medical care indicates a socio-economic gradient with the position of the lowest income group worsening. These findings are consistent with the expected impact of reducing equity of access to primary medical care and enabling more affluent residents to access additional and specialist services. We therefore urge careful consideration before the implementation of any measures likely to impede equitable access to health care.

### Ethics and permissions

The National Research and Development Centre for Welfare and Health Research Ethics Committee approved the research project, data protection measures were agreed with Statistics Finland, and the Finnish data protection ombudsman approved the data linkages.

## Competing interests

No competing financial interests.

AKM is the vice-chair of the CHI Advisory Group. This group was established by the Scottish Chief Medical Officer to advise Scottish Directors of Public Health on the appropriate use of the NHS Scotland unique personal identifier for secondary purposes. AKM is also a member of the UK Caldicott Guardians Council and has an interest in the appropriate use of individual level data for secondary purposes, including research. AKM is also asked to advise the Scottish Government Health and Justice Departments from time to time on matters relating to socio-economic inequality and the health of the population: regardless of the findings of this study, the outputs of this research programme would form part of that advice.

AHL is asked to advise the Chief Medical Officer of the Scottish Government from time to time on matters relating to socio-economic inequality and health and how to measure it: regardless of the findings of this study, the outputs of this research programme would form part of that advice.

IK is asked to advise the Finnish Ministry of Health and Social Affairs from time to time on matters relating to socio-economic inequality and health: regardless of the findings of this study, the outputs of this research programme would form part of that advice.

## Authors’ contributions

AKM participated in the study design and development, selection of variables, review and interpretation of the results, design and preparation of the manuscript. KM participated in the study design and development, guided the data analysis, interpretation of the results, and preparation of the manuscript. MA participated in the data preparation and analysis and drafting of the methods and findings sections of the manuscript. AHL participated in the study design and development, selection of variables, developed the models for analysis, guided the data analysis, participated in the preparation of the manuscript. IK participated in the study design and development, selection of variables, oversaw the data analysis and participated in the interpretation of the results, design and drafting of the manuscript. All authors read and approved the final manuscript.

## Pre-publication history

The pre-publication history for this paper can be accessed here:

http://www.biomedcentral.com/1472-6963/13/3/prepub

## Supplementary Material

Additional file 1: Table S1 Amenable mortality rates for 25-74 years old by income quintiles in 1992-2003. Annual total amenable mortality per 100,000 - Table of underlying data for Table S1. **Appendix 1**. Causes of death considered amenable to health care according to Nolte and McKee (modified). Click here for file
