# Supplementary material for: Socioeconomic differences in mortality amenable to health care among Finnish adults 1992-2003: 12 year follow up using individual level linked population register data
Source: BMC Health Serv Res. 2013 Jan 3;13:3. doi: 10.1186/1472-6963-13-3 (PMC3602718; doi:10.1186/1472-6963-13-3)
Supplement: Additional file 1: Table S1 — Amenable mortality rates for 25-74 years old by income quintiles in 1992-2003. Annual total amenable mortality per 100,000 - Table of underlying data for Table S1. Appendix 1. Causes of death considered amenable to health care according to Nolte and McKee (modified). [file 1472-6963-13-3-S1.doc]

# Figures: Figure 1 Amenable mortality rates for 25-74 years old by income quintiles in 1992-2003

Amenable mortality rates for 25-74 years old by income quintiles in 1992-2003. Annual total amenable mortality per 100,000 - Table of underlying data for Figure 1.

|  | Men | | | | | Women | | | | |
| --- | --- | --- | --- | --- | --- | --- | --- | --- | --- | --- |
|  | income quintile | | | | | income quintile | | | | |
|  | lowest | 2 | 3 | 4 | highest | lowest | 2 | 3 | 4 | highest |
| 1992 | 237.23 | 169.93 | 127.69 | 108.48 | 103.99 | 163.64 | 121.88 | 93.89 | 94.49 | 86.52 |
| 1993 | 241.00 | 173.67 | 117.88 | 111.01 | 98.72 | 170.10 | 126.96 | 117.98 | 93.60 | 82.69 |
| 1994 | 230.66 | 158.11 | 111.84 | 101.02 | 87.97 | 159.97 | 109.23 | 104.38 | 93.28 | 78.63 |
| 1995 | 258.32 | 162.03 | 127.78 | 99.71 | 85.26 | 180.11 | 121.04 | 107.97 | 92.40 | 82.49 |
| 1996 | 246.98 | 161.93 | 117.57 | 94.56 | 77.87 | 167.73 | 111.69 | 92.25 | 89.24 | 71.70 |
| 1997 | 252.82 | 161.55 | 109.93 | 93.33 | 72.34 | 183.67 | 116.22 | 99.74 | 85.08 | 70.39 |
| 1998 | 257.10 | 147.24 | 107.42 | 89.21 | 66.03 | 166.37 | 109.00 | 90.61 | 74.40 | 72.68 |
| 1999 | 243.02 | 147.15 | 94.77 | 103.73 | 68.28 | 167.27 | 114.18 | 95.08 | 66.45 | 67.12 |
| 2000 | 220.08 | 146.28 | 88.58 | 80.98 | 66.94 | 183.48 | 115.23 | 95.48 | 70.06 | 60.71 |
| 2001 | 208.24 | 131.24 | 86.12 | 81.08 | 65.40 | 169.54 | 104.55 | 73.20 | 70.32 | 60.78 |
| 2002 | 213.92 | 138.27 | 86.81 | 67.92 | 52.65 | 156.10 | 99.27 | 81.00 | 62.64 | 53.56 |
| 2003 | 212.96 | 128.52 | 89.19 | 66.32 | 50.98 | 147.71 | 89.17 | 75.08 | 66.01 | 53.85 |

Appendix 1. Causes of death considered amenable to health care according to Nolte and McKee (modified)

| | Timing and site of intervention | Name of group | Age | | ICD9 | | ICD10 | | --- | --- | --- | --- | --- | --- | --- | | Primary prevention | Intestinal infections | 1-14 | | 001-009 | | A00-A09 | |  | Other infections (Diphtheria, Tetanus, Poliomyelitis) | 1-74 | | 032, 037, 45 | | A36, A35, A80 | |  | Whooping cough | 1-14 | | 033 | | A37 | |  | Measles | 1-14 | | 055 | | B05 | | Early detection and intervention | Tuberculosis | 1-74 | | 010-018, 137 | | A15-A19, B90 | |  | Malignant neoplasm of colon and rectum(‡) | 1-74 | | 153-154 | | C18-C21 | |  | Malignant neoplasm of skin(‡) | 1-74 | | 173 | | C44 | |  | Malignant neoplasm of breast | 1-74 | | 174 | | C50 | |  | Malignant neoplasm of cervix uteri | 1-74 | | 180 | | C53 | |  | Malignant neoplasm of unspecified parts of uterus and body of uterus | 1-44 | | 179, 182 | | C54, C55 | |  | Hypertensive disease | 1-74 | | 401-405 | | I10-I13, I115 | |  | Cerebrovascular disease | 1-74 | | 430-438 | | I60-I69 | | Improved treatment and medical care |  |  | |  | |  | | -Predominantly primary care | Diseases of the thyroid | 1-74 | | 240-246 | | E00-E07 | |  | Diabetes mellitus | 1-49 | | 250 | | E10-E14 | |  | Epilepsy | 1-74 | | 345 | | G40-G41 | |  | All respiratory diseases (excl. pneumonia/influenza) | 1-14 | | 460-479, 488-519 | | J00-J09, J20-J99 | |  | Asthma (*) | 15-49 | | 493 | | J45, J46 | |  | COPD (*) | 15-49 | | 490-492, 496 | | J40-J44 | | -Predominantly specialist care | Septicaemia | 1-74 | | 038 | | A40-A41 | |  | Malignant neoplasm of testis(‡) | 1-74 | | 186 | | C62 | |  | Hodgkin’s disease(‡) | 1-74 | | 201 | | C81 | |  | Leukaemia | 1-44 | | 204-208 | | C91-C95 | |  | Chronic rheumatic heart disease (‡) | 1-74 | | 393-398 | | I05-I09 | |  | Influenza | 1-74 | | 487 | | J10-J11 | |  | Pneumonia | 1-74 | | 480-486 | | J12-J18 | |  | Peptic ulcer | 1-74 | | 531-533 | | K25-K27 | |  | Appendicitis | 1-74 | | 540-543 | | K35-K38 | |  | Abdominal hernia | 1-74 | | 550-553 | | K40-K46 | |  | Cholelithiasis & cholecystitis | 1-74 | | 574-575.1 | | K80-K81 | |  | Nephritis and nephrosis | 1-74 | | 580-589 | | N00-N07, N17-N19, N25-N27 | |  | Benign hyperplasia of prostate | 1-74 | | 600 | | N40 | |  | Maternal death | All | | 630-676 | | O00-O99 | |  | Perinatal deaths, all causes excluding stillbirths† | All | | 760-779 | | P00-P96, A33,A34 | |  | Congenital cardiovascular anomalies | 1-74 | | 745-747 | | Q20-Q28 | | -Ischaemic heart disease | Ischaemic heart disease | 1-74 | | 410-414 | | I20-I25 | |  | (‡) Condition not included as ‘amenable condition’ in Denmark, Finland and Sweden in Nolte & McKee's study | | | | | | |  | due to the classification of the WHO data based on ICD8 in these countries. Included in the REDD study | | | | | | |  | (*) Addition by McCallum | |  |  |  | | |  | (†) Not included in the REDD study | |  |  |  | | |
| --- | --- | --- | --- | --- | --- | --- | --- | --- | --- | --- | --- | --- | --- | --- | --- | --- | --- | --- | --- | --- | --- | --- | --- | --- | --- | --- | --- | --- | --- | --- | --- | --- | --- | --- | --- | --- | --- | --- | --- | --- | --- | --- | --- | --- | --- | --- | --- | --- | --- | --- | --- | --- | --- | --- | --- | --- | --- | --- | --- | --- | --- | --- | --- | --- | --- | --- | --- | --- | --- | --- | --- | --- | --- | --- | --- | --- | --- | --- | --- | --- | --- | --- | --- | --- | --- | --- | --- | --- | --- | --- | --- | --- | --- | --- | --- | --- | --- | --- | --- | --- | --- | --- | --- | --- | --- | --- | --- | --- | --- | --- | --- | --- | --- | --- | --- | --- | --- | --- | --- | --- | --- | --- | --- | --- | --- | --- | --- | --- | --- | --- | --- | --- | --- | --- | --- | --- | --- | --- | --- | --- | --- | --- | --- | --- | --- | --- | --- | --- | --- | --- | --- | --- | --- | --- | --- | --- | --- | --- | --- | --- | --- | --- | --- | --- | --- | --- | --- | --- | --- | --- | --- | --- | --- | --- | --- | --- | --- | --- | --- | --- | --- | --- | --- | --- | --- | --- | --- | --- | --- | --- | --- | --- | --- | --- | --- | --- | --- | --- | --- | --- | --- | --- | --- | --- | --- | --- | --- | --- | --- | --- | --- | --- | --- | --- | --- | --- | --- | --- | --- | --- | --- | --- | --- | --- | --- | --- | --- | --- | --- | --- | --- | --- | --- | --- | --- | --- | --- | --- | --- | --- | --- | --- | --- | --- | --- | --- | --- | --- | --- | --- | --- | --- | --- | --- | --- | --- | --- | --- | --- | --- | --- | --- | --- | --- | --- | --- | --- | --- | --- | --- | --- | --- | --- | --- | --- | --- | --- | --- | --- | --- | --- | --- | --- | --- | --- | --- | --- |
